# Surgery

**Published:** 1838-07

**Authors:** 


### SURGERY.

### *On Creosote, as a local Application.* By George Fife, m.d., Newcastle-upon-Tyne.

This communication contains ten cases of local disease, in all of which an ointment, composed of half a drachm or forty minims of creosote, and an ounce of lard or cerate, applied to or rubbed on the part affected, about twice daily, was productive of immediate and permanent relief. Four of the cases were of obstinate ulcer, in one of them assuming the form of lupus. "The above cases," says Dr. Fife, "will, I think, be received as tolerable evidence of the utility of creosote in not merely obstinate, but even malignant ulcers; and to these many others might be added. It is, however, only an useless expenditure of time to do so. I shall content myself with affirming, that in no case of ulceration in which I have yet tried it has it disappointed my expectations. I was first induced to try it from the perfect success attending its application to a case of cancer in the ear of a dog, about two years ago, a disease which I have no doubt many of the readers of the *Gazette* know to be most intractable; in fact, to defy every other means. In the case in question it was used in its concentrated state."

Two were cases of spontaneous neuralgia; two of neuralgic pains from severe sprain; one of the disease usually termed "rheumatic gout;" and one of ulcer of the throat. In this last case the creosote was used in the form of gargle. These cases are described with simplicity and commendable brevity, and deserve attention. *Med. Gazette. April* 7, 1838.

---

### *Case of Scalded Glottis, in which Laryngotomy was successfully performed.* By F. E. Hicks, Esq.

This case, as the author observes, "illustrates well the efficacy of calomel in scalded glottis, as recommended by Dr. Wallace, of Dublin, and also the necessity of sometimes assisting it by the operation of laryngotomy, without which it was clear in this case the calomel would have had no time to produce its effect."

On Thursday, March 1st, 1838, I was sent for by my friend Mr. Richardson, surgeon, of Bayswater, to see with him a little girl aged five years, who a few hours before had taken a tea-kettle of boiling water from off the fire, and drank from the spout a portion of its contents. At first the symptoms did not appear to be very urgent, but at the time I saw her the difficulty of breathing had become so alarming, owing to the inflammation and tumefaction which had taken place about the upper part of the larynx, as to threaten immediate suffocation; indeed, it was clear that unless something were done to relieve the breathing the child could not survive many minutes. I accordingly (assisted by Mr. Richardson) proceeded to perform the operation of laryngotomy, opening into the larynx in the usual situation between the thyroid and cricoid cartilages. The child being exceedingly restless, it was not thought right to introduce any tube into the opening; neither did we find that it was afterwards required, as our patient continued to breathe very freely through it for three or four days, when the air began to find its way through the natural channel, and it healed. For the first week or ten days the child refused to swallow any food, and required to be nourished with injections of broth and milk. Small doses of calomel, however, mixed with sugar, and placed dry in the mouth, appeared to make their way into the stomach, as on being repeated at short intervals for three or four days the system became mercurialized. Under this treatment the sloughs in the mouth rapidly separated, and at the expiration of a little more than three weeks from the accident, both the sores in the mouth, and the wound made by the operation, have healed, and the child is nearly restored to health.

*Med. Gazette. April* 7, 1838.

### *On some Effects resulting from Wounds of Nerves.*

By JOHN HAMILTON, L.R.C.S.

[THIS paper contains some interesting facts. It reclaims for the nerves what many modern surgeons have been disposed to attribute to the vessels. We have only space for one of the cases, and the conclusions drawn from the whole by Mr. Hamilton.]

CASE II. Catherine Cantwell, ætat. twenty, apparently in good health, but somewhat hysterical, came to me at the South Eastern Dispensary, with a wound in the palm of the hand. While contending with her brother for an apple, she went to give him a slap, when the palm of her hand came on the point of a knife, which entered at the middle of the ball of the thumb at its inner side, and nearly penetrated through. There was little bleeding, and the wound healed in a few days; but then a train of symptoms arose of a most troublesome character; the palm and back of the hand became greatly swollen, the swelling of a pale, œdematous character, exquisitely tender, and extremely painful; the tenderness was greatest about the cicatrix, and exceeded anything I had seen in other diseases. She got no sleep at night, when the pain was worse. At the same time a red line sprung up on the front of the foramen, at its external side, like that from inflamed lymphatics, but without the hard and knotty swelling. These symptoms were, for a time, diminished by antiphlogistic means, but recurred again and again; and about two months after the wound, the swelling got so red and large, and the parts about the wound assumed so much the appearance of a collection of matter, that I should have thought there was one, but that I had so often seen nearly all the swelling and redness disappear in a night, and after remaining away a few days, would re-appear at night, in the course of an hour or so, and for days assume a periodical character, increased and red towards evening, and during the night, and decreased and pale in the morning. The pain, if possible, became greater, the tenderness extreme, a sensation of pins and needles was felt in the hand and fingers, most so in the fore-finger and thumb, with pain and shooting up the arm, numbness, and feeling of deadness, and loss of power. The fingers and thumb were somewhat contracted, and could not be extended without great agony, nor could they be completely flexed, so as to hold any body firmly. She suffered also from hysterical symptoms, globus, palpitations, and low spirits. Having tried the usual routine of antiphlogistic remedies, general and local, as also, latterly, bark and other tonics, with nothing more than a very temporary alleviation, I was desirous of putting her on the use of mercury, but previous to doing so, showed her to Mr. Crampton. He agreed with me as to the symptoms being the result of a wounded nerve, and as to the propriety of administering mercury, suggesting, also, the local application of belladonna, which, however, had been used. Direct salivation was established, the happiest effects were manifest: the great pain and swelling went away, leaving slight tenderness, slight swelling, of a pale colour, on the back of the hand, and an occasional feeling of pins and needles. These were a long time before going off, three months after there being still the slightest possible œdema on the back of the hand, and a trifling degree of tenderness about the cicatrix and palm. She also complained that at night the pins-and-needles' sensation came on, and the hand and arm, always moist, then became covered with large beads of perspiration. After a time, these symptoms only recurred every second or third night; but, though able to follow her usual employment at the end of nine months, it was at least a year before she was quite well.

From the cases here given, and many others that I do not wish to tire my readers with, I feel myself justified in stating, that a wound of a nerve, or any of its branches in peculiar states of the constitution, may be followed by most distressing local and constitutional symptoms, viz.:

1. Pain of the severest character in the injured part, or shooting from it in various directions in the course of the nerves to the extremities, or to the brain. That it is often periodical, and generally attended with the most exquisite tenderness.

2. That with the pain there is frequently redness and swelling, resembling a good deal the appearance of the skin in inflammation of the fascia or a deep collection of matter, but differing, in being of a paler colour and more œdematous character, in being subject to sudden increase and diminution, and at times being only periodical.

3. Contraction of the limb, and spasms of an unusually violent character; the spasms in some instances going into the genuine convulsion of epilepsy.

4. That the constitutional symptoms are those commonly called nervous and hysterical, viz.: depression of spirits, prostration of strength, globus, &c., but that hectic fever has also presented itself.

5. That all the symptoms, uninfluenced by every remedy made use of, have gradually declined after a long interval, having apparently undergone a natural cure; but that in a few instances the termination has been fatal.

It is not easy to account for these symptoms being present in only a few cases of wounded nerve; for in the greater number of injuries daily occurring, in which the nerves must be wounded, they do not ensue. They are most frequently observed in nervous and hysterical women, but not always.

*Dublin Journal of Med. Science. March,* 1838.

---

### *Case of Talipes Varus Verus, affecting both Limbs.*
By W. J. Little, m.d., *London.*

[This is a striking example of the great value of the operation revived by Dr. Stromeyer, and so successfully practised by Dr. Little and other surgeons in this country. The patient was a lad, aged sixteen, and the deformity was congenital. The following extracts give the more important parts of the case, and also show Dr. Little's mode of operating.]

*Condition of the Patient when seen by Dr. Little, (April* 5*th,* 1837.) The first circumstance which strikes the observer is the imperfect development of the whole organization of the lower extremities, which are disproportionately short, compared with the length of the trunk of the body, and with that of the upper extremities; the muscles of the pelvis and thighs are, although well cased in adipose tissue, much below the usual standard, while those of the leg are still less developed; indeed, there is a total absence of the calves of the legs from atrophy of the gastrocnemii. The shortness of each limb depends less upon deficiency of the femur than of the tibia and fibula; the shafts of the latter can also be distinctly felt to be more slender than natural. The feet are also stunted in their growth; both heels are held up two inches from the ground by the shortness of the gastrocnemii, and the toes are drawn inward, and the inner margins of the feet upwards, by the shortness of the tibialis posticus, so that the great toes are in contact with one another, and the patient touches the ground by that part only of the plantar surfaces of the feet, called the ball of the little toe. This part, which is an oval of an inch and a half in length, is covered by a painful corn, through having had to bear all the weight of the body. Of the remainder of the soles of the feet, the ball of the great toe, the heel and under part of the instep, no part has ever come into contact with the earth. There is very little mobility of the anklejoints, particularly of the left, scarcely sufficient to enable the surgeon to render the Achilles' tendon tense; the ligamentous tissues are very rigid, and those of the inside of the feet (lig. deltoidea), as the form of the feet indicates, are particularly contracted; the legs are both œdematous, and the skin purplish, from languor of the circulation in them; their temperature, also, is unusually low.

The patient's mode of progression, when he has boots on, which, having heels and broad soles, are accommodated to his lameness, is extremely laborious and difficult; his arms maintain the equilibrium of his body, as without useful gastrocnemii, and with so extremely small a point of support upon the earth as is afforded by the ball of the little toes, he would be in constant danger of falling were it not from the agility the constant practice from infancy has afforded him; his arms are almost constantly in motion, until his body has found the perpendicular; like the dancer upon the tight or slack rope, he raises the one foot over the other alternately, giving the limb, whilst it is moving, a peculiar semicircular swing, first outwards, then forwards, and, lastly, inwards. Without his boots, unaided by the arm of his mother, or by two sticks, he cannot make a single step. The peculiar swing I have described, arises from the endeavour to avoid touching the toe of one foot against those of its fellow, and to bring as large a part of the sole against the ground as possible; for if such a patient were to place his feet perpendicularly on the ground, the point of the foot would turn gradually still more inwards, and the ankle outwards, the tendency of which would be to cause him to tread upon the dorsum pedis, in which case, although the deformity is increased, progression is less laborious to the patient.

*Operation.* April 11, 1837. I divided both the Achilles' tendons. The knife used is a very narrow, slightly curved, sharp-pointed bistoury, the blade one line in width at the widest part, and an inch and a half long, ground to a slightly concave cutting edge, in half only of its length, the remaining half (that connected with the handle) being left blunt. The operation I perform as follows,—the patient being seated in a chair, one assistant supports the knee firmly, whilst another, grasping and drawing downwards the patient's heel with his left hand, and pressing upwards the toes and front of the foot with his right, renders the tendon to be divided as tense as possible. After feeling with my left thumb and forefinger the outline of the tendo Achillis (enveloped in this patient in much adipose tissue, owing to which no relief of the tendon was visible externally), I pass the small bistoury through the skin, one or two fingers' breadth, above the malleolus internus, with one of its sides turned towards the tendon and immediately above it (the patient sitting), the other directed towards the deeper muscles and the tibial vessels and nerves; as soon as I know that the point of the knife has been passed beyond the external edge of the tendon, and has nearly reached the skin of the opposite side, I turn the knife so as to bring its cutting edge to press against the tendon, which is divided generally at one stroke, in the act of withdrawing the knife from the limb. The complete division of the tendon is known by the sensation of the immediate cessation of the tense resistance, and by feeling, before the knife is wholly withdrawn, that nothing remains undivided except the flaccid integuments. (The operation does not occupy a quarter of a minute.) After the withdrawal of the knife, there remains a single puncture in the skin of less than a line in length, from which seldom oozes more than a single drop of blood; such was the case in both legs in the present instance. A few strips of adhesive plaster, and a roller, were applied, and each leg and foot secured to a pasteboard splint, adapted to their deformed shape, to prevent motion of the joint, as it is a wise principle of the Stromeyerian method to make no attempt to draw down the heel, or improve the form of the foot (which would be prematurely interfering with the effusion of lymph between the ends of the tendons, and with the closure of the external punctures), until the latter heals, which it uniformly does by first intention.

Everything proceeded favorably, and the latest report, dated, September 1, 1837: At the present time, he informs me that he can walk about all day with comfort, ten or twelve miles without difficulty, in the capacity of errand-boy. It could not be known that he had formerly had two congenital club-feet; the only remains of them are a slight turning in of the toes when he is fatigued, and, consequently, careless about his gait, and an evident want of the natural freedom of action in the ankle-joints. The mass of muscle has evidently increased. He did not wear the Scarpa shoes after the tenth week, and has worn a common pair of lace-up boots ever since, stiffened on the inner side of the ankle with an additional piece of leather.

[In three subsequent Nos. of the Lancet (March 31, May 19 and 26,) there are three more cases of distortion by Dr. Little. The cure in all was equally prompt and complete, and by the same means.]

*Lancet. March* 17, 1838.

*Remarks on Lenticular Glaucoma.* By WILLIAM MACKENZIE, M.D., Surgeon-Oculist to the Queen, for Scotland.

THE object of this paper, which is written with the clearness and deep knowledge of the subject, observable in all the author's communications on diseases of the eye, is to prove, contrary to the general opinion of the most distinguished oculists, "that by far the greater number of glaucomata depend essentially on the state of the lens." "I am inclined to think (says Dr. Mackenzie) that there is never any very distinct glaucomatous appearance (that is to say, cloudiness of a greenish hue,) except what is caused by the amber or reddish-brown colour of the central and posterior laminæ of the lens. In lenticular glaucoma, the lens has become, in a certain sense, dichromatic, being of a deep amber when allowed to transmit the light, but appearing green by reflected light; the green hue being probably the result of the absorption of the extreme prismatic rays of the light entering the eye, while the middle prismatic rays are but little affected."

The author also shows that the *catoptrical examination* of the eye, in the manner proposed by M. Sanson, (noticed at p. 231 of our present Number,) "confirms, in the most satisfactory manner, the doctrine that glaucoma is, in general, an affection of the crystalline lens." *Med. Gazette. April* 14, 1838.

---

*A Mode of relieving Patients labouring under Enlargement of the Veins of the Testicle.* By THOMAS WORMALD, Esq.

WHEN cases of varicocele are allowed to proceed without any active means being adopted for their relief, the patients may experience much inconvenience from pain in the loins and spermatic cord, and frequently are incapacitated from walking any considerable distance.

P. W., aged nineteen, applied to me in the year 1832, in consequence of a circocele of very large dimensions, which had existed two years, and had been progressively getting worse. The veins were distended to the size of a large apple; so much was he inconvenienced, that a walk of half a mile produced great pain in the back and spermatic cord. After a consultation with Sir A. Cooper, cold lotions, suspensory bandages, &c., having been employed without affording the slightest relief, Sir Astley recommended the removal of a portion of the scrotum. To this proceeding the patient would not consent. I therefore adopted the following mode of treatment:—A ring, about an inch in diameter, made of soft silver wire, of a suitable thickness, was padded, and covered with wash-leather. Through this I drew the lower part of the scrotum, whilst the patient was in the recumbent position, and the veins comparatively empty. I then pressed the sides of the instrument towards each other with sufficient force to prevent the scrotum escaping. The use of this instrument every morning before the patient rose from his bed, enabled this gentleman to walk nineteen miles on the third day after the first application; and although he has for six years worn an instrument of this description, he has never experienced the least inconvenience. And I may add, that other patients (and amongst them medical friends) labouring under varicocele, have found the greatest relief from this simple contrivance.

*Med. Gazette. April* 28, 1838.

---

*Treatment of Intussusception by Inflation.* By S. MITCHELL, Esq. Surgeon, Kingston.

[ALTHOUGH this is not a new practice, it deserves notice; the more so as we know that it has succeeded in other instances.]

The case presented all the usual symptoms: intolerable restlessness, the most obstinate sickness, the singularly distressed state of countenance and shrunken features. The usual remedies were had recourse to,—viz. warm baths, clysters, anodyne frictions over the abdomen, &c.; but without avail. As a forlorn hope, I made trial of inflation by means of a clyster-pipe attached to a common pair of bellows, with the most happy result: the sickness immediately ceased, the child within an hour passed a natural stool, fell into a sleep, and in the morning was almost without ailment. *Lancet. March* 17, 1838.

